# The CRISPR tool kit for genome editing and beyond

**DOI:** 10.1038/s41467-018-04252-2

**Published:** 2018-05-15

**Authors:** Mazhar Adli

**Affiliations:** Department of Biochemistry and Molecular Genetics, School of Medicine, University of Virginia, 1340 Jefferson Park Ave, Pinn Hall, Rm: 640, Charlottesville, VA 22902 USA

## Abstract

CRISPR is becoming an indispensable tool in biological research. Once known as the bacterial immune system against invading viruses, the programmable capacity of the Cas9 enzyme is now revolutionizing diverse fields of medical research, biotechnology, and agriculture. CRISPR-Cas9 is no longer just a gene-editing tool; the application areas of catalytically impaired inactive Cas9, including gene regulation, epigenetic editing, chromatin engineering, and imaging, now exceed the gene-editing functionality of WT Cas9. Here, we will present a brief history of gene-editing tools and describe the wide range of CRISPR-based genome-targeting tools. We will conclude with future directions and the broader impact of CRISPR technologies.

## Abstract (web summary)

CRISPR has rapidly become an indispensable tool for biological research. Here Mazhar Adli reviews the current toolbox for editing and manipulating the genome and looks toward future developments in this fast moving field.

## Introduction

Great inventions and discoveries are often storied as a series of lucky coincidences. However, a closer look at their history reveals that truly serendipitous discoveries are very rare, if not absent in molecular biology. This is perhaps true for other scientific disciplines too. Groundbreaking scientific advancements have several characteristics. They are often built on decades of combined efforts of many great minds. Even so-called serendipitous discoveries come when an inquisitive and open-minded researcher designs a series of careful experiments to follow an interesting observation. During this process, researchers with creative minds and deep background knowledge can seize the opportunity to converge seemingly separate research fields and make a bigger scientific impact. The genome-editing technologies and CRISPR tools have come to the current exciting stage through years of basic science research and progress from a large number of researchers. This review will present the brief history and key developments in the field of genome editing and major genome-engineering tools. However, for the most part the review will focus on the CRISPR technology. The application areas of CRISPR technology that are extending beyond genome editing, such as targeted gene regulation, epigenetic modulation, chromatin manipulation, and live cell chromatin imaging, will be particularly emphasized. Finally, it will briefly discuss current and future impacts of these tools in science, medicine, and biotechnology.

## Brief history of genome-editing efforts

Genomes of eukaryotic organisms are composed of billions of DNA bases. The ability to change these DNA bases at precisely predetermined locations holds tremendous value not only for molecular biology, but also for medicine and biotechnology. Therefore, introducing desired changes into genomes, i.e., “genome editing”, has been a long sought-after goal in molecular biology. To this end, the discovery of restriction enzymes that normally protect bacteria against phages in the late 1970s^1–3^ was a turning point that fueled the era of recombinant DNA technology. For the first time ever, scientists gained the ability to manipulate DNA in test tubes. Although such efforts drove a number of discoveries in molecular biology and genetics, the ability to precisely alter DNA in living eukaryotic cells came a few decades later. To this end, several key developments were revealed in the mid to late 1980s. Initial targeted gene disruption studies in eukaryotic yeast cells^4^ followed with breakthrough work by Capecchi and Smithies in mammalian cells^5–7^. Their studies demonstrated that mammalian cells can incorporate an exogenous copy of DNA into their own genome through a process called homologous recombination^5–7^. Such targeted gene integration into the genome provided unprecedented power to characterize the functional roles of various genes in model organisms. However, the feasibility of this approach had several limitations. Firstly, the rate of spontaneous integration of an exogenous DNA copy was extremely low (1 in 10^3^–10^9^ cells)^7^. Secondly, the integration rate depended on cell types and cellular states. Finally, and most critically, the approach could result in random integration of the exogenous copy into undesired genomic loci at a frequency similar to or higher than that of the target site^8^.

## Development of targeted nucleases for genome editing

Researchers sought alternative approaches to overcome these aforementioned limitations. One of the initial breakthroughs came from the realization that the introduction of a double-strand break (DSB) at a target site results in a several orders of magnitude increase in the frequency of targeted gene integration^9,10^. Therefore, many research groups focused on developing different strategies to achieve targeted DSBs. In the early studies, researchers utilized rare cutting endonuclease enzymes, such as the 18-bp cutter **I-SceI**, to introduce specific DSBs in the mouse genome^10^. Although such meganucleases (the endonucleases that recognize long stretches of 14–40 bp DNA) increased the genome-editing efficiency, the approach was restricted by two major drawbacks. Firstly, despite the presence of hundreds of naturally found meganucleases, each of them has a unique recognition sequence. Thus, the probability of finding a meganuclease that targets a desired locus was still low. Secondly, and more critically, the majority of induced DSBs are repaired through the error-prone non-homologous end joining (NHEJ) DNA repair mechanism. Thereby, not only may the exogenously introduced DNA template not incorporate at the DSBs, but also the NHEJ repair mechanism may randomly insert or delete DNA pieces at the break sites^11^. To overcome such challenges, researchers started to re-engineer naturally existing meganucleases to alter their DNA-targeting specificities^12–14^. Although these efforts significantly improved the possibilities of targeted editing, only a very small fraction of genomes could be specifically targeted using meganucleases.

**Fig. 1:**
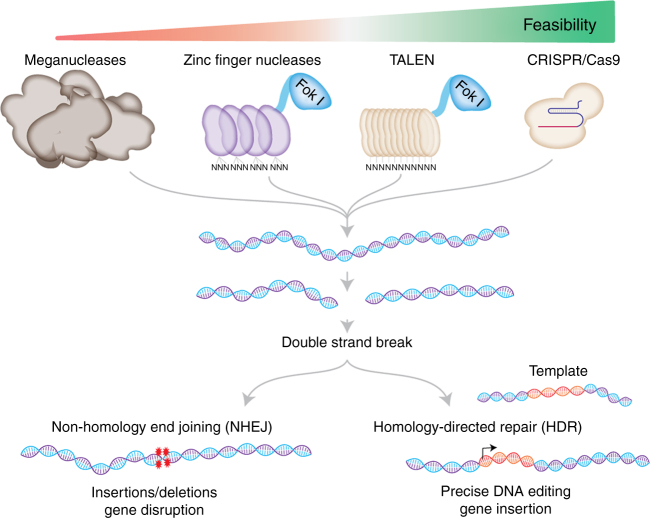
The basic working principle of major genome-editing technologies. Meganucleases are engineered restriction enzymes that recognize long stretches of DNA sequences. Each zinc finger nuclease recognizes triple DNA code whereas each TALE recognizes an individual base. Unlike protein–DNA recognition in ZFNs and TALENs, simple RNA–DNA base pairing and the PAM sequence determine CRISPR targeting specificity. All these tools result in DNA double-strand breaks, which are repaired either by error-prone non-homology end joining or homology-directed repair. While NHEJ results in random indels and gene disruption at the target site, HDR can be harnessed to insert a specific DNA template (single stranded or double stranded) at the target site for precise gene editing

To this end, the discovery and utilization of eukaryotic zinc finger proteins started a new era in genome targeting and editing. Zinc fingers are zinc ion-regulated small protein motifs that bind to DNA in a sequence-specific manner. Each zinc finger module recognizes a 3-bp DNA sequence^15^. Therefore, unlike meganucleases, multiple zinc finger modules could be assembled into a larger complex to achieve higher DNA binding specificity. Soon after the structure of zinc fingers was revealed, researchers started to create programmable nuclease proteins by fusing zinc finger proteins with the DNA cleavage domain of the **Fok I** endonuclease^16^. The choice of the Fok I restriction enzyme was a well-considered, deliberate choice for couple of reasons. Firstly, unlike many other restriction enzymes, Fok I has distinct DNA recognition and DNA cleavage domain. Knowing this, researchers removed the DNA sequence recognition domain of Fok I and fused only the DNA cleavage domain to zinc finger protein modules. Another critical consideration is that Fok I requires homodimerization at the target site to cleave DNA. Therefore, designing two separate zinc finger modules that target two proximal sites next to each other allows Fok I to homodimerize and result in DNA strand breaks at the target sites. The zinc finger nucleases (ZFNs) were shown to significantly increase targeted homologous recombination not only in model organisms but also in human cells^17,18^. The improved efficiency in the design of ZFNs tremendously enhanced the capabilities to edit genomes of living cells at specifically targeted sites and opened doors for therapeutic applications of such genome-editing tools^19,20^. Since each zinc finger recognized a 3-bp DNA code, combinatorial assembly of 6–7 zinc fingers out of the unique 64-finger pool (4^3^ combinations) could uniquely target any 18–21 bp genomic sequence^21^. While ZFNs generated substantial excitement as a genome-engineering tool, the discovery that transcription activator-like effector (TALE) proteins from *Xanthomonas* bacteria can specifically recognize one single base instead of three bases has inspired further excitement about these proteins^22,23^. Like zinc fingers, chimeric fusion of the Fok I DNA cleavage domain to a combination of TALE modules serves as an effective programmable nuclease, called a TALEN^24–27^ (Figure 1).

## The rise of CRISPR as the genome-editing technology

Although the discovery of artificially designed meganucleases followed by ZFNs and TALENs successively increased the genome-editing efficacy, targeting different sites in the genome required re-design or re-engineering of a new set of proteins. The difficulty in cloning and protein engineering ZFNs and TALENs partially prevented these tools from being broadly adopted by the scientific community. In this respect, CRISPR has revolutionized the field because it is as robust as, if not more so than, the existing tools in terms of editing efficiency. More importantly, it is much simpler and more flexible to use. The CRISPR gene-editing technology, as we know it today, is composed of an endonuclease protein whose DNA-targeting specificity and cutting activity can be programmed by a short guide RNA. Notably, CRISPR had been simply known as a peculiar prokaryotic DNA repeat element for several decades before it was recognized as the bacterial immune system and subsequently harnessed as a powerful reprogrammable gene-targeting tool.

**Fig. 2:**
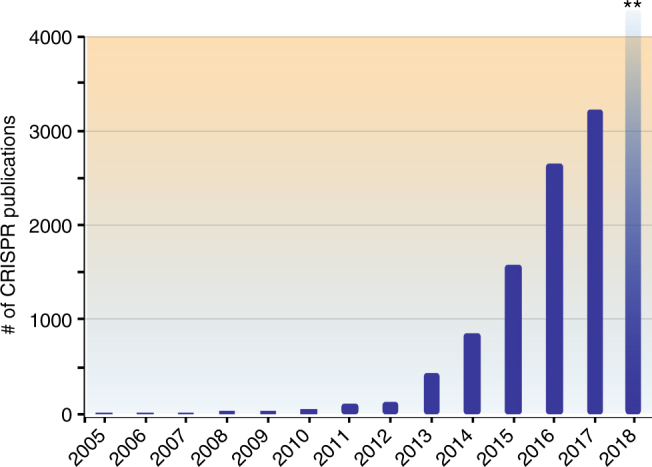
CRISPR-based genome-targeting tools are widely used. Number of PubMed publications over the last 12 years that had the word “CRISPR” or “Cas9” in the abstract or title. **Number of publications in 2018 is projected to be more than 5000

CRISPR stands for clustered regularly interspaced short palindromic repeat DNA sequences. Although the name CRISPR was coined much later^28^, these repeat elements were initially noticed in *Escherichia coli* by Dr. Nakata’s group^29^. Interestingly, unlike typical tandem repeats in the genome, the CRISPR repeat clusters were separated by non-repeating DNA sequences called spacers. It took more than a decade for researchers to recognize the nature and origin of these spacer sequences. During the human genome project (HGP), the genomes of many other organisms, including many different phages, were also sequenced. The computational analysis of these genomic sequences led researchers to notice key features of CRISPR repeat and spacer elements. Firstly, the CRISPR sequences are present in more than 40% of sequenced bacteria and 90% of archaea^30^. Secondly, the CRISPR elements are adjacent to multiple well-conserved genes called CRISPR-associated (Cas) genes^28^. Finally and most interestingly, the non-repeating spacer DNA sequences were recognized to belong to viruses and other mobile genetic elements^31–33^. These observations sparked the interest of many researchers in studying the functional significance and mechanics of these CRISPR sequences. Although the idea that it could serve as a bacterial immune system started to circulate among researchers^31,32,34^, the exact mechanism of action was not known. The key experimental evidence about the potential function of CRISPR systems came from the work of Horvath and colleagues. They demonstrated that after a viral challenge, *Streptococcus thermophilus* bacteria integrate new spacers derived from the phage genomic sequence into its genome. More importantly, the spacer sequences of CRISPR dictate the targeting specificity of Cas enzymes, which provide defense against the phage^35^. Immediately following this work, other researchers further elucidated the mechanism of action of the CRISPR system. Within a year after this key discovery, it was shown that the activity of Cas enzymes is guided by short CRISPR RNAs (crRNA) transcribed from the spacer sequences^36^ and that it can block horizontal DNA transfer from bacterial plasmids^37^. Such exciting publications further stimulated researchers’ interest in understanding the molecular mechanism of the CRISPR system. There have been several critical findings that paved the way for CRISPR systems to become the CRISPR genome-editing technology. One of the key findings was the observation that the acquired spacer sequences are highly similar to each other at regions called protospacer-adjacent motifs (PAMs) and that this sequence is very critical for the CRISPR system to work^38^. Independently, it was revealed that among many Cas proteins, Cas9 was the only one with DNA catalytic activity in *S. thermophilus*^39^. Additionally, the work from the Charpentier group revealed the mechanism of biogenesis of the two short RNAs required for Cas9 action^40^. A final critical discovery was the demonstration that a CRISPR system from one bacterium was transferable to different bacterial strains. Siksnys and colleagues showed that the CRISPR locus from *S. thermophilus* is able to reconstitute the interference in *E. coli*^41^. These findings were immediately followed by biochemical characterization of the individual components of the CRISPR system. The crucial work, which arguably marked the beginning of CRISPR as a biotechnology tool, has been the demonstration that Cas9 enzymes can be reprogrammed to target a desired DNA sequence in bacteria^42,43^. Notably, these studies also simplified the CRISPR system by using a single short RNA. The endogenous CRISPR system requires two short RNAs: the mature crRNA and a trans-activating crRNA (tracrRNA). The crRNA is composed of the part that serves as guiding sequence and another part base pairs with the tracrRNA. Both crRNA and tracrRNAs are required to form the Cas9 protein–RNA complex that cleaves DNA with DSBs at target sites. Notably, Jinek et al. showed that CRISPR-Cas9 can also be guided by a single chimeric RNA formed by the fusion of tracrRNA and crRNA, called single guide RNA (sgRNA)^42^. These studies were immediately followed by groundbreaking publications showing that CRISPR can be adapted for in vivo genome editing in eukaryotic cells^44–46^. For the first time ever, researchers had an extremely flexible tool that could be easily guided to target nearly any location in the genome by simply designing a short sgRNA. Due to high editing efficiency and ease of use, researchers from diverse fields quickly adopted CRISPR technology as a method of choice for various genome-targeting purposes. Notably, since its inception as a genome-editing tool in late 2012, more than 9000 research articles have been published about it and the number of publications seems to continue to increase each year (Fig. 2).

## Different CRISPR systems and their uses in genome editing

The evolutionary arms race between prokaryotes and environmental mobile genetic elements such as phages has been going on for billions of years. This survival struggle yielded various CRISPR-type immune responses as defense mechanisms in bacteria. These CRISPR systems are classified based on the structure of CRISPR-associated (Cas) genes that are typically adjacent to the CRISPR arrays^47,48^. The classification efforts are yet to be completed as researchers continue to discover new systems and refine the classification system with subclasses, groups, and types based on comparative genomic analyses, structures, and biochemical activities of CRISPR components^49^. Broadly speaking, there are two classes of CRISPR systems, each containing multiple CRISPR types. **Class 1** contains type I and type III CRISPR systems that are commonly found in Archaea. **Class 2** contains type II, IV, V, and VI CRISPR systems^49^. Although researchers repurposed many different CRISPR/Cas systems for genome targeting, the most widely used one is the type II CRISPR-Cas9 system from *Streptococcus pyogenes*. Because of the simple NGG PAM sequence requirements, *S. pyogenes’* Cas9 (spCas9) is used in many different applications. However, researchers are still actively exploring other CRISPR systems to identify Cas9-like effector proteins that may have differences in their sizes, PAM requirements, and substrate preferences. In the last few years, more than 10 different CRISPR/Cas proteins have been repurposed for genome editing (Table 1). Among these, some of the recently discovered ones, such as Cpf1 proteins from *Acidaminococcus sp* (AsCpf1) and *Lachnospiraceae bacterium* (LbCpf1), are particularly interesting^50–52^. In contrast to the native Cas9, which requires two separate short RNAs, Cpf1 naturally requires one sgRNA. Furthermore, it cuts DNA at target sites 3′ downstream of the PAM sequence in a staggering fashion, generating a 5′ overhang rather than producing blunt ends like Cas9 (Table 1).

**Table 1:** Naturally occurring major CRISPR-Cas enzymes

	Size	PAM sequence	Size of sgRNA guiding sequence	Cutting site	Reference
spCas9	1368	NGG	20 bp	~ 3 bp 5′ of PAM	Jinek et al.^42^
					Gasiunas et al.^43^
FnCas9	1629	NGG	20 bp	~ 3 pb 5′ of PAM	Hirano et al.^60^
SaCas9	1053	NNGR RT	21 bp	~ 3 pb 5′ of PAM	Mojica et al.^57^
NmCas9	1082	NNNNG ATT	24 bp	~ 3 bp 5′ of PAM	Hou et al.^53^
St1Cas9	1121	NNAGA AW	20 bp	~ 3 bp 5′ of PAM	Gasiunas et al.^43^
					Cong et al.^45^
St3Cas9	1409	NGGNG	20 bp	~ 3 bp 5′ of PAM	Gasiunas et al.^43^
					Cong et al.^45^
CjCas9	984	NNNNACAC	22 bp	~ 3 bp 5′ of PAM	Kim et al.^56^
AsCPf1	1307	TTTV	24 bp	19/24 bp 3′ of PAM	Yamano et al.^50^
					Kim et al. 2016
LbCpf1	1228	TTTV	24 bp	19/24 bp 3′ of PAM	Yamano et al.^50^
					Kim et al. 2016
Cas13	Multiple orthologs	RNA targeting	28 bp		Abudayyeh et al. 2017

Naturally found Cas9 variants are large proteins, which adds particular limitation when it comes to their packaging and delivery into different cell types via Lenti or Adeno Associated viruses (AAV). For example, the widely used SpCas9 protein is 1,366 aa, which creates a particular therapeutic delivery challenge due to the limited packaging capacity of AAV. Thus, smaller Cas9 variants have greater therapeutic potential. To this end, the discoveries of 1082 aa Cas9 from Neisseria meningitides (NmCas9)^53^, 1053 aa Cas9 from *Staphylococcus aureus* (SaCas9)^54,55^, and 984 aa Cas9 from *Campylobacter jejuni* (CjCas9)^56^ are major forward steps toward this goal. However, the tradeoff is that these smaller Cas9 proteins require more complex PAM sequences. The SaCas9 requires a 5′-NNGRRT-3′ PAM sequence^54,55,57^ whereas CjCas9 requires a 5′-NNNNACAC-3′ PAM sequence^56^. Therefore, these smaller Cas9 proteins have relatively limited targeting scope and flexibility in genome targeting compared to SpCas9 despite the reduction in size.

## Re-engineering CRISPR-Cas9 tools

Exploring different CRISPR systems requires extensive understanding and characterization of new Cas proteins. Thus, in parallel to these studies, there are increasing efforts to re-engineer the already well-characterized Cas9 proteins. This research direction is focusing on achieving three major goals: (i) reducing the size of Cas9 nucleases, (ii) increasing their fidelity, and (iii) expanding the targeting scope of Cas9 variants. Although there has been a limited advance in reducing the size of existing Cas9 proteins, several groups have altered the Cas9 PAM requirements and targeting specificity. In one such study, researchers used an unbiased selection strategy to identify variants of SpCas9 and SaCas9 with more relaxed PAM sequence requirements^58,59^. In line with these findings, a different study utilized a structure-guided design strategy to re-engineer FnCas9 to recognize YG PAM sequences instead of NGG^60^.

In addition to these studies that expand the targeting scope of CRISPR tools, researchers are actively developing novel ways to increase the targeting specificity of the CRISPR-Cas9 system. Understanding the extent of off-target effects of CRISPR-Cas9 targeting has been one major goal. Given that CRISPR systems have evolved as a defense system against viruses that tend to frequently mutate, a slightly less specific CRISPR system would be advantageous to bacteria. Indeed, the early efforts to understand CRISPR targeting specificity highlighted this fact and demonstrated that the system may potentially have off-target effects^61–65^. In addition to these initial studies, researchers utilized alternative genome-wide tools to understand CRISPR-Cas9 targeting specificity. To this end, we and others have used the chromatin immunoprecipitation and high throughput sequencing (ChIP-Seq) approach to map DNA binding sites of catalytically inactive SpCas9 in vivo^66,67^. These whole-genome mapping studies highlighted that Cas9 off-target binding sites are enriched at open chromatin regions. The analysis of SpCas9 binding sites together with chromatin accessibility data (DNase-Seq) across 125 different human cell types demonstrated that integrating chromatin state data enables better in silico prediction of Cas9 off-target binding sites^68^. Notably, detailed analyses of off-target bindings indicated that the system allows a number of mismatches at PAM distal sites. However, only limited numbers of the off-target binding sites were cleaved in vivo, indicating a less stringent requirement for Cas9-DNA binding versus DNA cleavage^66,67^. Since Cas9 binding does not necessitate DNA cleavage, alternative approaches have been taken to study genome-level DNA cleavage specificity of Cas9 variants. Although whole genome deep sequencing can potentially identify indels due to DSB, associated sequencing, and analytical costs, researchers developed BLESS^69^, GUIDE-Seq^70^ and Digenome-Seq^71^ approaches to specifically enrich the sites that undergo DSB. Detailed comparative analyses of these different mapping approaches are beyond the scope of this review, however it is important to note that each approach has its own unique advantages and limitations. Therefore, it remains a challenge to truly determine an inspection process that maps all of the CRISPR-Cas9-mediated DNA cleavage and binding sites, as these can be dependent on sgRNA guiding sequences, the cell type, and sgRNA/Cas9 delivery methods.

In parallel to these approaches to assess the off-target effects of the system, several forward steps have been taken to increase the targeting specificity of CRISPR-Cas9 systems by re-engineering the existing spCas9 variants. In one study, researchers identified specific point mutations that significantly increase the specificity of SpCas9^72^. Similarly structure-guided rational designs resulted in Cas9 variants with enhanced targeting specificity^73^. In addition to such re-engineering efforts on the Cas9 structure, researchers are utilizing alternative targeting approaches to substantially reduce the off-target binding and cleavage activity of Cas9. One of the easiest ways to increase the targeting specificity is changing the delivery method of the Cas9-sgRNA complex. In contrast to plasmid-based delivery, direct delivery of Cas9-sgRNA as a ribonucleotide protein (RNP) complex results in more transient Cas9 activity and hence less off-target effects^74,75^. Additionally, tandem targeting a locus with two separate sgRNAs utilizing either the nickase Cas9 (nCas9)^62,76^ or catalytically inactive Cas9 (dCas9)^77,78^ fused to the DNA cleavage domain of the Fok I substantially reduces the off-target activity of WT Cas9. Since these approaches require two separate guide RNAs to be in a certain proximal distance, the probability of off-target modification is substantially reduced. In parallel to these approaches, inducible Cas9 approaches using small molecule chemicals^79^, optical light^80,81^, and ligand-dependent allosteric regulation^82^ to control temporal and spatial activities of the Cas9/sgRNA complex have also improved targeting specificity. In addition to such engineering approaches at the Cas9 protein, efforts also focused on modifying the sgRNA scaffold to increase the targeting specificity. Interestingly, both increasing^65^ and decreasing^83^ the length of the sgRNA guiding sequence by a few base pairs have been reported to enhance the targeting specificity. Furthermore, incorporating ligand-responsive self-cleaving catalytic RNAs (aptazymes) into guide RNA may allow temporal control over the targeting activities of the CRISPR-Cas9 complex^84^.

## Utilizing CRISPR-Cas9 beyond genome editing

**Fig. 3:**
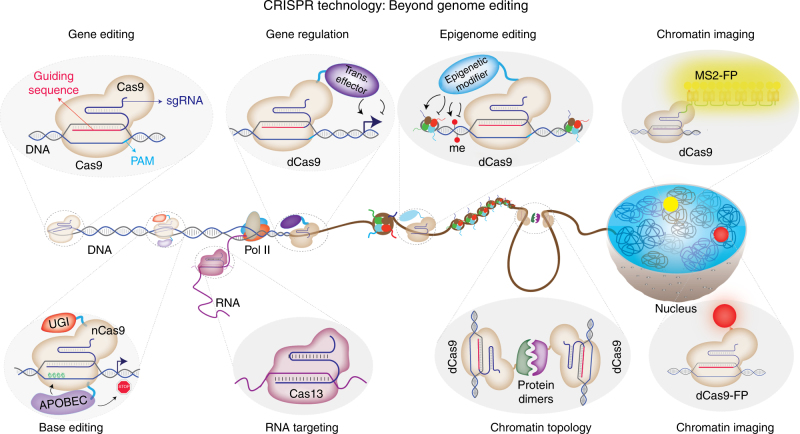
Major application areas of CRISPR-Cas-based technologies beyond genome editing. While WT Cas9 enables genome editing through its guidable DNA cleavage activity, catalytically impaired Cas9 enzymes have been repurposed to achieve targeted gene regulation, epigenome editing, chromatin imaging, and chromatin topology manipulations. Furthermore, the catalytically impaired nickase Cas9 enzyme has been used as a platform for base editing without double strand breaks. In addition to DNA-targeting Cas proteins, novel RNA-targeting CRISPR/Cas systems have been described as well

So far, the review has focused on the basic mechanism of CRISPR targeting and some of the recent approaches that have been utilized to monitor or improve the targeting specificity of CRISPR-Cas9. Due to its robustness and flexibility, CRISPR is becoming a versatile tool with applications that are transforming not only genome-editing studies, but also many other genome and chromatin manipulation efforts. As summarized in Fig. 3, these alternative application areas are largely possible because of the programmable targeting capacity of catalytically inactive dead Cas9 (dCas9)^85^, which cannot cleave DNA but can still be guided to the target sequence^42^. CRISPR-Cas9 has two catalytic domains (HNH and RuvC) that act together to mediate DNA DSBs^86^. Each of these catalytic domains cleaves one DNA strand, thereby resulting in DSBs proximal to the PAM sequence at the target site. Notably, a single point mutation in either of these domains results in a nickase enzyme, whereas mutations in both domains (D10A and H840A for SpCas9) results in complete loss of DNA cleavage activity^42^. Researchers have repurposed these Cas9 variants for a wide range of genome-targeting purposes. As previously noted, tandem targeting of nickase Cas9 has been utilized to improve targeting specificity^62,76^.

## Evolution of second-generation CRISPR gene-editing tools

One of the key progresses in the field of CRISPR technology has been the development of base-editing technology. Unlike WT Cas9, which results in DSBs and random indels at the target sites, these so-called second-generation genome-editing tools are able to precisely convert a single base into another without causing DNA DSBs. The nickase Cas9 is the foundational platform for the base editor tools that enables direct C to T or A to G conversion at the target site without DSBs^87–89^. Komor et al. recently demonstrated that a fusion complex composed of nickase Cas9 fused to an APOBEC1 deaminase enzyme and Uracyl Glycosylase inhibitor (UGI) protein effectively converts Cytosine (C) into Thymine (T) at the target site without causing double strand DNA breaks^88^. Notably, a transfer RNA adenosine deaminase has also been evolved and fused to nickase Cas9 to develop another novel base editor that achieves direct A–G conversion at the target sites^87^. These novel base-editing approaches significantly expand the scope of genome targeting. Researchers are further developing these tools for additional purposes. We, and others, recently harnessed the efficiency of this CRISPR base editor to alter genetic code and introduce early STOP codons in genes^90,91^. We show that by editing C into T at CGA (Arg), CAG (Gln), and CAA (Gln) codons, we can create TGA (opal), TAG (amber), or TAA (ochre) STOP codons, respectively. The CRISPR-STOP approach is an efficient and less deleterious alternative to WT-Cas9-mediated gene knockout (KO) studies^91^. In addition to the APOBEC adenosine deaminase enzyme, the activation-induced adenosine deaminase (AID) enzyme has also been fused to the dCas9 enzyme^92,93^. Notably, in the absence of UGI in the complex, the dCas9–AID complex becomes a powerful local mutagenic agent that acts as a gain of function screening tool^92–94^. For further details about various applications of CRISPR base-editing tools, please refer to review articles that comprehensively cover these novel application areas.

## CRISPR-mediated gene expression regulation

Soon after the initial demonstration that WT Cas9 can be used as a programmable endonuclease for gene editing, researchers started to exploit dCas9 to specifically regulate gene expression. In the following sections, we will highlight some of the application areas where researchers are uniquely repurposing dCas9 for various regulatory purposes (Fig. 3). Interestingly, dCas9 strongly binds to the DNA target sequence and this tight binding interferes with the activity of other DNA binding proteins such as endogenous transcription factors and RNA Polymerase II^85^. This has been exploited to develop the CRISPR interference (CRISPRi) approach in which dCas9 binding activity blocks the transcriptional process and thus knocks down (KD) gene expression^85^. Notably, fusing a strong repressor complex such as Kruppel-associated Box (KRAB) to dCas9 results in a stronger and more specific gene repressor than dCas9 alone^95^. The repression module of the KRAB protein, which is present in a large fraction of human zinc finger transcription factors, is ~45 amino acid (a.a). The KRAB-containing zinc finger proteins constitute the largest family of transcriptional repressors in mammals^96^. These transcriptional regulators further recruit additional co-repressor proteins such as KRAB-box-associated protein-1 (KAP-1) and epigenetic readers such as heterochromatin protein 1 (HP1) proteins to repress genes^97^. The KRAB-mediated gene repression is partly mediated by local epigenetic reprograming of histone modifications. It has been shown that KRAB-mediated gene repression is associated with loss of histone H3-acetylation and an increase in H3 lysine 9 trimethylation (H3K9me3) at the repressed gene promoters^98^. In line with these findings, the dCas9-KRAB fusion complex results in reduced chromatin accessibility and increased H3K9me3 levels at both targeted gene promoters as well as distal enhancers^99^.

**Fig. 4:**
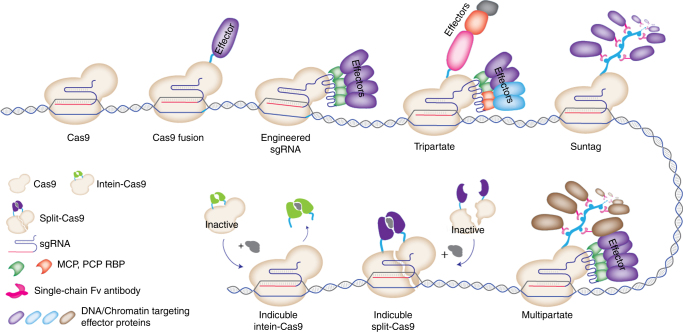
Major strategies to recruit DNA- and chromatin-targeting and modifying enzymes via the CRISPR-Cas system. The schematics show various strategies of recruiting effector proteins to a target site using RNA guidable DNA binding capacity of Cas9-sgRNA complex. Effector proteins can be directly fused to active Cas9 or catalytically inactive dCas9 through a linker peptide. Additionally, the sgRNA scaffold can be engineered to contain multiple RNA aptamers that specifically bind to a known RNA binding proteins (RBP) such as MCP or PCP. Effector proteins than can be guided to a target locus by fusing them to the RBPs. In the Tripartite strategy, multiple different effectors are being recruited through dCas9 as well as engineered sgRNA scaffold. The SunTag approach utilizes a repeating peptide array of protein scaffold to recruit multiple copies of an antibody-fused effector protein. Chemically inducible strategies enable temporal control over the activity of Cas9 or Cas9 fused effector proteins. In split Cas9, each halves of Cas9 protein can be induced to form functional complex. In the intein-Cas9 approach, the intein protein segment can be chemically induced to excise from Cas9 and result in its activation

In contrast to dCas9-KRAB-mediated gene repression, using the dCas9-targeting platform to recruit strong transcriptional activators results in robust induction of gene expression. To this end, the initial studies fused dCas9 to VP64^62,100,101^, which is composed of four tandem copies of a 16-amino-acid-long transactivation domain (VP16) of the Herpes simplex virus^102^. The dCas9-VP64-mediated gene activation strategy was further improved by a number of second-generation CRISPR-based gene activation platforms. In addition to fusing dCas9 with various copies of the VP16 protein^103^, researchers also fused dCas9 to a tripartite transactivation complex, which is composed of VP64, P65, and Rta (VPR) proteins, to achieve robust gene induction (Fig. 4)^104^. P65 is a transcription activation domain of the mammalian NF-κB transcription factor, whereas Rta is an R transactivator (Rta) from the Epstein-Bar virus^105^. Notably, in addition to directly fusing to dCas9, the effector domains can also be recruited through the sgRNA scaffold. For these approaches, the sgRNA scaffold is engineered to contain RNA modules such as MS2 hairpin aptamers that can bind to specific RNA binding proteins such as bacteriophage MS2-coat protein (MCP)^106,107^. In one such study, researchers used the engineered sgRNA-MS2 scaffold to recruit MCP-fused VP64^108^ or the P65-HSF1 transactivation complex (HSF1: heat shock transcription activator)^109^ to activate expression from an endogenous locus. In another approach called a synergistic activation mediator (SAM) complex, in addition to dCas9-VP64 fusion complex, MCP-fused P65-HSF1 transactivation domains were recruited to the target site through the engineered sgRNA scaffold^109^. Additionally, in a novel strategy termed SunTag, dCas9 fused protein scaffold that contains repeating peptide array has been used to recruit multiple copies of an antibody fused effector protein^110^. Now that various approaches are available for locus-specific gene induction, a practical challenge is to figure out which approach is ideal. The answer is likely cell type and context dependent. However, recent comparative analysis of various dCas9-based gene activation strategies across multiple species (several human, mouse, and fly cell lines) concluded that the VPR, SAM, and Suntag systems are consistently superior to the standard VP64 standard^111^. Furthermore, although SAM approaches show a trend of higher potency, these approaches had comparable efficiencies within an order of magnitude difference in fold change of locus-specific gene expression induction^111^. Inducing expression of endogenous loci has many advantages over exogenous expression. Precise spatial and temporal control over the dynamics of gene expression from a target locus has great therapeutic potential. The flexibility of the CRISPR approach enabled researchers to adopt various ways to achieve this. Inducible CRISPR targeting through optogenetics and small molecules are among the more notable advances within the CRISPR-mediated gene expression approaches^112–115^.

## CRISPR-mediated epigenome editing

The definition of “epigenetics” is heavily debated^116,117^. Here, we use the word “epigenetic” to imply the molecular mechanism of heritable gene expression changes that cannot be attributed to changes in DNA sequence information. Unlike epigenetics, which implies the mechanism, the epigenome describes all post-translational modifications and other chromatin features associated with regulatory elements in the genome. Recent large-scale epigenomic efforts such as the Encyclopedia of DNA elements (ENCODE) and Roadmap Epigenome Mapping Consortium (REMC) efforts have mapped chromatin modifications both on DNA and histone proteins across the genome in various cell lines as well as primary cell types and tissues^118,119^. Although these epigenomic maps revealed unprecedented insight into cell-type-specific gene regulation and genome organization, the functional roles of various epigenomic features, such as histone modifications and DNA methylation, remain to be fully understood. To this end, locus-specific epigenome mapping tools and technologies are expected to greatly empower researchers to elucidate functional roles of chromatin modifications. Such tools will enable investigating some of the long-standing questions of chromatin biology such as the causal relationship between the presence of an epigenetic mark and gene expression. Furthermore, the ability the alter locus-specific epigenetic marks may enable us to identify the temporal kinetics of an epigenetic mark and its physical role on the functional epigenetic memory and gene expression. Therefore, soon after the CRISPR-Cas9 system was harnessed as an efficient gene-editing technology, researchers used the programmable capacity of dCas9 to recruit various epigenetic writers and erasers to a specific locus.

There are multiple layers of epigenetic regulatory mechanisms operating in the genome. Among the well-described ones are DNA methylation, histone posttranslational modifications, and non-coding RNAs (short and long). Among these, DNA methylation has the longest history, as researchers noticed and started to study its role in gene expression and development in the early 1970s^120,121^. DNA methylation is one of the most widely studied epigenetic mechanisms of gene regulation. Notably, in plants and other organisms, DNA methylation is found in three different sequence contexts: CG (or CpG), CHG, or CHH (H is A, T, or C), whereas in mammalian systems, the majority of DNA methylation happens at the fifth carbon of Cytosine residues (5-methylcytosine) of CpG dinucleotides^122^. DNMT3A and DNMT3B are the two DNA methyltransferase enzymes that catalyze de novo DNA methylation^123^. 5-Cytosine DNA methylation at promoter or distal regulatory elements is generally associated with transcriptional repression. Aberrant DNA methylation has been implicated in a number of pathological diseases including cancer. Therefore, there is strong unmet therapeutic need to manipulate aberrant disease-associated epigenomic features. In line with this, some of the small molecule epigenetic inhibitors that globally target DNA methylation such as 5-azacytidine are FDA approved^124^. Although such small molecules are already in clinical use, they target the entire genome and thus alter the chromatin state of loci where the epigenetic state is normal. Therefore, developing locus-specific epigenetic editing tools that specifically target aberrantly regulated loci has great therapeutic potential. To achieve this proof of principal, researchers utilized the dCas9 system to both deposit DNA methylation marks as well as remove the endogenous DNA methylation from the target site. To deposit DNA methylation at a specifically targeted locus, researchers fused dCas9 to the catalytic domain of eukaryotic DNA methyl transferase (DNMT3A)^125–132^ or prokaryotic DNA methyltransferase (MQ3)^129^. In both strategies, substantial deposition of DNA methylation and altered gene expression were observed at the target site. Importantly, targeted recruitment of additional components of repressive epigenetic machinery such as KRAB-ZNF, DNMT3L and polycomb complexes further enhanced the robustness of DNA methylation and long-term sustained gene repression^126,133^. These early proof of principle studies have reported highly specific deposition of DNA methylation at the target loci and local effects on gene expression. Interestingly, by using a DNA methyltransferase-deficient embryonic stem cell model, a recent study reported that dCas9-fused DNA methyl transferase has global off-target effects by leaving methylation footprints that are independent of sgRNA and methods of delivery^134^. Notably, despite the global increase in DNA methylation, which was attributed to abundant free nuclear dCas9 fused methyl transferase; limited impact on gene expression was observed^134^. It remains to be seen whether reducing the total free dCas9-fused methyl transferase will be as efficient and whether this pervasive global off-target effect is also a general characteristic of other epigenetic effectors. In addition to targeted DNA methylation, active removal of local methylation marks from endogenous loci is another strategy to manipulate gene expression through DNA methylation. Endogenous DNA demethylation is carried out by ten-eleven translocation (TET) proteins: TET1, TET2, and TET3. The proteins play a critical role in dynamic epigenetic regulation that mediates cell type-specific gene expression programs and lineage specification during development^135^. Therefore, a number of research groups aimed to achieve locus-specific DNA demethylation by using guidable dCas9 as a platform to recruit the catalytic domains of TET proteins^125,131,132,136^. Notably, the dCas9-TET1 fusion complex resulted in DNA demethylation in up to 90% of local CpG dinucleotides and a substantial increase in mRNA expression at the target sites^125,131,132^. Although robust locus-specific DNA demethylation and altered gene expression on target sites were reported, it remains to be seen whether dCas9-TET fusions may leave a global demethylation footprint akin to the methylation footprint of dCas9-fused methyltransferase.

In addition to DNA methylation, epigenetic information is stored in histone proteins, which DNA is wrapped around to form the chromatin fiber. Posttranslational modifications on histone tails constitute major epigenomic features that reveal key insights about regulatory activity of genomic elements. For example, active distal regulatory elements in the genome are marked with mono- and di-methylation at the lysine four position of Histone H3 (H3K4me1/2) and acetylation at the Lysine 27 acetylation position (H3K27ac), whereas active or poised bivalent promoters are marked with tri-methylation of Lysine four (H3K4me3)^137^. Chromatin modifications are dynamically regulated by various epigenetic writers, readers, and erasers^138^. Therefore, researchers are exploiting the versatile dCas9 platform to recruit various histone modifiers to a specific locus to better study the downstream effects of histone modifications. Research in this area has been focused on locally depositing histone methylation or acetylation as well as removing such marks. It should be noted that, unlike histone methylation, which could be associated with active and repressive chromatin features depending on the site of methylation, histone acetylation is observed at active gene promoters and enhancers.

In one study, researchers targeted a dCas9-LSD1 fusion complex to manipulate the regulatory activity of distal enhancer regions^139^. LSD1 is a histone demethylase that removes H3K4me2 mark^140^. In line with previous TALE-based LSD1 fusion studies^141^, dCas9-mediated locus-specific recruitment of LSD1 resulted in substantial local reduction in the active enhancer markers H3K4me2 and H3K27ac^139^ and altered expression of target genes. Active enhancer elements are marked by both H3K27ac as well as H3K4me1/2. Therefore, an alternative approach to epigenetically manipulating enhancer function is to locally deposit H3K27ac marks. Therefore, in contrast to the local reduction of enhancer marks by dCas9-LSD1, recruitment of histone acetyl transferase P300 through dCas9 fusion (dCas9-P300) resulted in a significant increase in local H3K27ac levels at enhancer elements^142^. Importantly, unlike other dCas9-fused transactivators, which can result in induction of gene expression primarily from promoter regions, targeting dCas9-P300 allows significant gene expression induction from both promoter and enhancer regions^142^. Researchers have also exploited other epigenetic modifiers to manipulate additional epigenetic marks. Among these, dCas9 fusion to the PRDM9 methyltransferase fusion complex has been utilized to manipulate local H3K4me3 marks^143^. Notably, local induction of H3K4me3, which is a marker of active promoters, was observed to be sufficient to allow re-expression of silenced target genes in various cell types^143^. Histone de-acetylation has been another strategy to locally manipulate chromatin structure and function. To this end, dCas9 fusion to histone deacetylases (HDAC), specifically full-length HDAC3, has been shown to effectively reduce the H3k27ac at the target loci and reduce the gene expression of the target loci^144^.

These aforementioned locus-specific epigenetic manipulation strategies are based on overexpression of a dCas9-fused epigenetic modifier complex. Such tools have been shown to specifically manipulate the expression of the target loci. However, whether overexpression of the fusion epigenetic complexes may leave a low level but global epigenetic footprint in the genome, as noted for the dCas9–DNMT3A fusion complex^134^, is yet to be determined. Therefore, novel strategies that enable local recruitment of endogenous epigenetic machineries may provide a higher precision in epigenetic editing. To this end, novel approaches such as Fkbp/Frb-based inducible recruitment for epigenome editing by Cas9 (FIRE–Cas9)^145^ may provide higher specificity in epigenetic editing by recruiting endogenous chromatin regulators.

Identifying the causal link between epigenetic marks and gene expression remains a central goal of chromatin biology. Thus, these aforementioned studies using dCas9 as a guidable platform to edit locus-specific epigenetic information will be an indispensable tool to achieve this. Now that the tools that enable us to alter the epigenome are in place, the next phase is to utilize them to better characterize regulatory elements and cellular states. To this end, researchers have already applied dCas9-based epigenome-editing tools for a number of exciting purposes including high-throughput screenings to characterize functional distal enhancers^146^, targeted reprogramming of lineage specification^147,148^, generation of induced pluripotent stem cells^149^, and reversal of HIV latency^150^. One of the remaining challenges is to elucidate the causal relationship between the presence of an epigenetic mark and its regulatory impact. Since the dCas9-fused epigenetic modifier remains associated with the target site, it is unclear whether the regulatory activity is due to the induced epigenetic mark or the complex. To this end, recent efforts using rapid and reversible epigenome-editing approaches are highly notable^145^. Future studies that enable rapid degradation of the targeting complex at the target site, such as with auxin-inducible degron technology^151^, should allow us to further characterize the functional consequences of epigenetic marks and investigate the associated temporal epigenetic memory for each mark.

## CRISPR-mediated live cell chromatin imaging

The organization of chromatin structure in the 3D nuclear space plays a critical role in regulating lineage-specific gene expression^152^. Historically, fluorescent in-situ hybridization (FISH) methods have been fundamental in determining the precise nuclear positions of specific genetic loci^153–155^. However, inherent limitations, such as the requirement of cell fixation and sample heating, prohibited the application of this tool to live cell imaging. Previously, researchers used zinc fingers (ZNF)^156^ and TALE proteins^157^ for targeted recruitment of fluorescent proteins to repetitive genomic regions, such as centromeres and telomeres for live cell imaging. However, the advances in the dCas9 platform technology have substantially improved both the efficiency and scope of genome targeting for live cell chromatin imaging. Researchers used fluorescently labeled dCas9 to target repetitive regions of the genome to achieve the goal^158^. A similar approach has been utilized to target repetitive natures of telomeres and centromeres by co-expression of dCas9 orthologs fused to different fluorescent proteins^159,160^ and dual-color chromatin imaging of these repetitive regions^160–163^. Targeting dCas9 to a non-repetitive genomic locus is more challenging because of the background fluorescence signals due to free-floating fluorescently labeled dCas9 proteins. Therefore, transfection of as many as 26–36 unique sgRNAs is typically required to achieve live cell imaging of a non-repeat genomic region^158,164^. To overcome this challenge, we recently utilized engineered sgRNA scaffolds which contains up to 16 MS2 binding modules to enable robust fluorescent signal amplification and allow imaging a repeat genomic region with as few as 4 sgRNAs^165^. The engineered sgRNAs enabled multicolor labeling of low-repeat-containing regions using a single sgRNA and of non-repetitive regions with as few as four unique sgRNAs^165^. Notably, this approach enabled tracking of native chromatin loci throughout the cell cycle and determining differential positioning of transcriptionally active and inactive regions in the 3D nuclear space^165^.

## CRISPR-mediated manipulation of chromatin topology

Another exciting area of CRISPR applications to chromatin biology is directed engineering of chromatin loop structures. Targeted engineering of artificial chromatin loops between regulatory genomic regions provides a means to manipulate endogenous chromatin structures to understand their function and contribution to gene expression. Such efforts may enable the formation of new enhancer–promoter connections to overcome certain genetic deficiencies. Additionally, an aberrantly active enhancer–promoter interaction can be inhibited. Thus such efforts have great therapeutic potentials too. In this respect, the demonstration that gene expression can be induced from a developmentally silent endogenous locus through forced chromatin looping was a significant step forward in demonstrating the potential for this system^166^. Researchers are now using dCas9-based platforms to achieve targeted and robust manipulation of chromatin structure and DNA loop formation. In an elegant recent study^167^, Morgan et al. took advantage of two dimerizable protein domains (ABI1 and PYL1) from the plant-based abscisic acid (ABA) signaling pathway^168^. Tethering these protein-dimerization systems, to two separate dCas9 orthologous, enabled forced chromatin loop formation between distal enhancer and promoter regions. Notably, this inducible chromatin loop resulted in increased gene expression at the β-globin locus in the relevant K562 hematopoietic cells but not in HEK293T cells^167^. In an independent study, researchers utilized strong heterodimerizing leucine zippers to target dCas9 orthologs and achieve targeted DNA looping in a prokaryotic system^169^. These proof of principle studies demonstrate the power of CRISPR as a targeted chromatin structure-rewiring tool. These tools are expected to play critical roles in pushing the frontier of synthetic chromatin biology.

## Large-scale genetic and epigenetic CRISPR screenings

In addition to targeted genetic and epigenetic manipulations, the simple and efficient gene-targeting capacity of CRISPR has been harnessed to achieve large-scale functional screenings. In such applications, instead of using a single sgRNA, WT Cas9 or dCas9-effector fusion proteins are guided with hundreds or thousands of individual sgRNAs in a population of cells. The ultimate aim for such studies is to identify genes that influence a specific phenotype in an unbiased fashion^170–172^. Although the approach requires a number of technical and analytical considerations^173^, once established, such an approach becomes a powerful high-throughput assay to functionally screen a large number of genes at the same time. In its basic form, a large pool of Cas9/sgRNAs are typically delivered to a population of cells via a low multiplicity of viral infection (MOI = 0.3 to 0.4). This ensures that each cell is receiving one or less sgRNA. For robust statistical readouts, each gene is typically targeted by 6–10 different sgRNAs. The basic logic behind the CRISPR KO screenings is that if a gene is essential for a given phenotype, such as cell proliferation, then the cells infected with the sgRNAs targeting that gene will be relatively depleted from the population over time. Since each sgRNA is stably integrated into the genome during viral infection, the guiding sequences of each sgRNA can be used as a unique ‘barcode’. Thus the relative abundance of each sgRNA in a given population of cells can be quantified by targeted sequencing. The specific details of such assays are beyond the scope of this review. The in-depth technical and analytical details as well as the wide range of CRISPR screening applications have been excellently covered in other review articles^173^.

## Future directions

Development of novel tools and technologies is indispensable for scientific advancement. Nobel laureate Sydney Brenner is quoted as saying “Progress in science depends on new techniques, new discoveries and new ideas, probably in that order”^174^. Surely, CRISPR-based technologies have empowered researchers with an unprecedented toolbox. The history of molecular biology will place CRISPR-Cas9 among the major tools that enabled breakthrough discoveries and methodological advancements in science. CRISPR applications have already expanded our vision of genome regulation and organization in living cells across diverse biological kingdoms. In this regard, CRISPR is not only transforming molecular biology but also medicine and biotechnology. Due to space limitations, this review only focused on the major CRISPR tools. However, several multiple recent review articles have comprehensively overviewed the specific applications of CRISPR tools^173,175–181^. Within the last few years we have witnessed stunning progress in the development of various CRISPR-based technologies. The therapeutic applications of the CRISPR technologies are particularly exciting^182^. Such advancements have been widely covered in social and other mass media outlets, inspiring great excitement and interest from the general public. However, the rapid development of CRISPR-based tools also brings forth a number of technical challenges along with social and ethical concerns.

One of the technical challenges is the delivery of such tools into living cells and organisms. Researchers commonly use viral vectors to deliver genes of interest in vivo or in vitro. Due to their low immunogenicity, AAV vectors are particularly attractive therapeutic delivery vehicles for in vivo settings. However, the large size of current Cas proteins creates a major challenge in their packaging into AAV vectors. Therefore, future advancements in reducing the size of existing Cas proteins or the discovery of smaller Cas9 proteins is highly needed. As CRISPR technologies grow in scope and power, social and ethical concerns over their use are also rising, and applications of these powerful tools deserve greater considerations^183^. One such CRISPR application with a long-lasting outcome is the so-called “gene drive” that can potentially target an entire population or a species^184^. In this powerful CRISPR application, researchers have demonstrated that a gene allele that provides parasite-resistant phenotype in mosquitos can quickly spread through the population in a non-Mendelian fashion^185,186^. Such applications may greatly empower us in the war against malaria-type diseases. However, due to the global effect of such applications, safety backups should be carefully designed and additional regulatory procedures should be considered and implemented in advance^187,188^.

The CRISPR-based technologies will undoubtedly continue to transform basic as well as clinical and biotechnological research. However, the road ahead is not free of obstacles. One such obstacle is the potential immunogenicity to CRISPR-Cas9 proteins. The most widely used Cas9 proteins are from *S. aureus* and *S. pyogenes*. Notably, since these bacteria cause infectious disease in humans at high frequencies, a recent report documented that more than half of humans may already have pre-existing humoral and cell-mediated adaptive immune responses to Cas9 proteins. Therefore, as the CRISPR-Cas9 system moves forward into clinical trials, this factor must be taken into account^189^. Studying and understanding such challenges will enable us to better determine the scope of their limitations and ways to overcome them. To this end, one proposed solution to the immunogenicity problem could be to identify and utilize orthogonal CRISPR-Cas9 proteins to which we as humans have not been introduced before^190^. It is likely that many more naturally occurring CRISPR systems will be discovered and that they will be harnessed for additional genome-targeting platforms. Therefore, in parallel to the current advancements, additional studies are needed to address the safety and specificity of such tools. Furthermore, sufficient considerations need to be devoted to the social and ethical implications of such technologies so that they will be accessible to all layers of society and benefit all humankind.
